# Magnetic Characterization in the Rayleigh Region of Nanocrystalline Magnetic Cores

**DOI:** 10.3390/ma11112278

**Published:** 2018-11-14

**Authors:** Mikel Osinalde, Pablo Infante, Lurdes Domínguez, Juan Mari Blanco, Alexander Chizhik, Valentina Zhukova, Arcady Zhukov, Julian González

**Affiliations:** 1 R&D Department, ELESA Transformadores S.A., 20800 Zarauz, Gipuzkoa, Spain; mosinalde@elesa-trafocore.com (M.O.); pinfante@elesa-trafocore.com (P.I.); 2 Department of Applied Physics I, University of the Basque Country, Plaza Europa s/n, 20018 San Sebastián, Spain; marialourdes.dominguez@ehu.eus (L.D.); juanmaria.blanco@ehu.eus (J.M.B.); 3 Department of Materials Physics, Faculty of Chemistry, University of the Basque Country, Paseo Manuel de Lardizabal 3, 20018 San Sebastian, Spain; valentina.zhukova@ehu.es (V.Z.); arkadi.joukov@ehu.eus (A.Z.); julianmaria.gonzalez@ehu.eus (J.G.); 4 IKERBASQUE, Basque Foundation for Science, 48011 Bilbao, Spain

**Keywords:** magnetic characterization, Finemet, Rayleigh region

## Abstract

We report on the structural and magnetic characterization of two nanocrystalline Finemet-type magnetic cores. The nanocrystalline structure developed after annealing the amorphous precursor alloy at 550 °C for 30 and 60 min of annealing time. Structural analysis carried out by means of X-ray diffraction providing useful information on the grain size mean and partial volume of the nanocrystalline phase. The magnetic characterization was focused mainly in the Rayleigh region which, influenced by the intergranular coupling, was found to be more efficient in the sample treated for a longer time with a finer nanocrystalline structure.

## 1. Introduction

Nowadays, nanocrystalline magnetic cores are essential components in modern technologies, since they are used in a wide range of highly efficient commercial products in many electrical and electronic devices operating at low or medium frequencies [1,2,3]. In spite of the knowledge gathered on soft magnetic nanocrystalline alloys, the development of ultrafine grain structures with outstanding magnetic properties still displays puzzling aspects. With respect to this, emergent applications of magnetic cores involve them to be used in the so called Rayleigh region, that is, the magnetization curve at a small exciting magnetic field. In addition, the reduction of electric energy consumption of new electronic devices is a challenge for their strategic implications. In this context, the magnetic behavior of soft magnetic materials in the aforementioned Rayleigh area can provide useful information strongly connected with aspects on energy because such behavior is quite different on comparing with the magnetization curve at high magnetic fields [4,5].

It is well known that the outstanding soft magnetic character of nanocrystalline alloys (Finemet-type) is strongly connected with their peculiar microstructural features. In fact, such nanocrystalline structure is obtained on submitting the precursor amorphous alloy to careful thermal treatment, obtained by non-equilibrium techniques involving a rapid solidification process. The thermal treatment (typically at 550 °C, 60 min.) provokes a massive nucleation and growth of α-Fe or α-Fe(Si) nanograins (10–20 nm of diameter) embedded in the residual amorphous matrix. Obtained in this way, nanocrystalline alloys present excellent magnetic softness, quite low coercivity (*H_C_*) values and magnetic losses as well as enhanced initial magnetic permeability values [6,7,8]. It is noteworthy that post-thermal treatment as well as the chemical composition of the precursor alloy can be careful selected allowing the possibility of tuning the properties of nanocrystalline materials considering each particular application [9,10]. As a very interesting consequence to mention, these soft nanocrystalline materials are ideal candidates to be used as the nucleus of small electrical motors.

Additionally, research on the magnetic behavior of soft magnetic materials with amorphous and nanocrystalline structure in the Rayleigh region can provide useful information regarding these applications. In this context, previous works (see for example [10,11,12]) analyzed the magnetization process in the Rayleigh region of these materials as one part of the complete magnetization process until the magnetic saturation state, a few of these focused only on the deep analysis of such a region [13].

In this paper we present a microstructural study carried out by means of X-ray diffraction (XRD) as well as experimental results concerning the magnetic behavior in the Rayleigh region in toroidal wound cores of Fe_73.5_Cu_1_Nb_3_Si_15.5_B_7_ amorphous alloy annealed at 550 °C for 30 and 60 min, which induced a massive nanocrystallization. Therefore, our aim was to achieve a deeper understanding of the magnetic characterization in the so-called Rayleigh region (at low applied magnetic field) in this interesting new magnetic material within its nanocrystalline state as nucleus of a toroidal magnetic core.

## 2. Experimental Details

Fe_73.5_Cu_1_Nb_3_Si_15.5_B_7_ amorphous material ribbons (width: 10.3 mm, thickness: 18 µm) produced by the melt-spinning technique were used to build a nucleus of the toroidal wound core, built with ring-shape geometry (external diameter = 54.5 mm, inner diam. = 45.7 mm, bandwidth = 10.3 mm). Then, the nucleus was submitted to thermal treatment (550 °C for 30 and 60 min) in a specific furnace (Brockhaus Messtechnik, Plettenberg, Germany). These two thermal treatments provoked the development of the nanocrystalline structure as was evidenced by XRD analysis (Bruker AXS GMBH, Karlsruhe, Germany). From here on, the nanocrystalline core treated for 30 min of duration is denoted as core A and that treated for 60 min as core B.

Wide angle X-ray scattering was used in a powder diffractometer to characterize the microstructure. The measurements were carried out using the step scanning technique between 40° and 80° (2θ) in steps of 0.02° (2θ) with accumulation times of 3 s at each point. Note that 2θ is the scattering angle and the region of measurement is the one where these compositions show their more significant crystalline peaks with CuKα wavelength.

Magnetic parameters in the Rayleigh region of the core tape were performed through the virgin magnetization curve of the nanocrystalline magnetic cores measured from the hysteresis loop at room temperature and at low applied magnetic field (up to 50 A/m). For this, we used hysteresis graph equipment (MPG 100 D model from Brockhaus Messtechnik, Plettenberg, Germany) in quasistatic conditions (more details can be found in Reference [1]).

## 3. Results and Discussion

**Figure 1:**
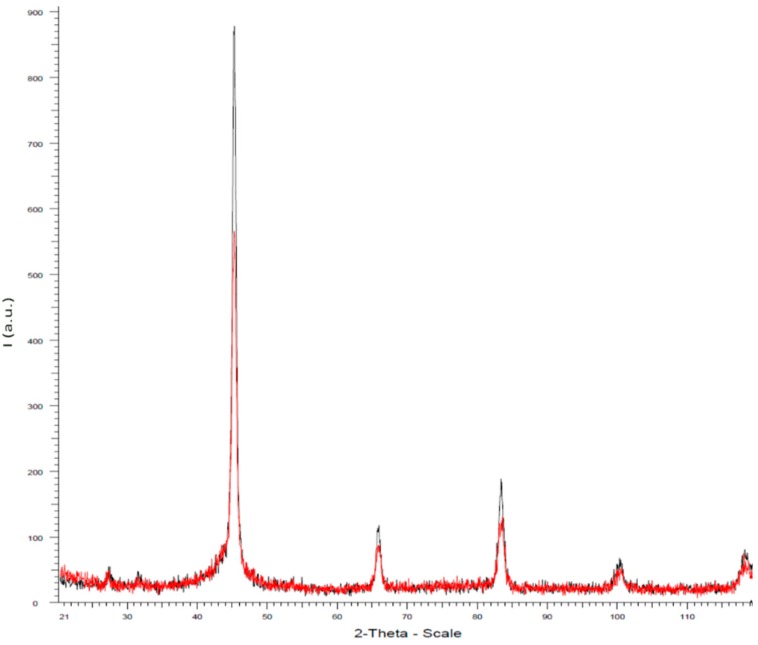
XRD scans obtained for the two nanocrystalline samples: A (red) and B (black) core.

Figure 1 depicts the XRD patterns of pieces of the two nanocrystalline magnetic cores. Diffraction patterns were normalized in the high *q*-range where the intensity cannot vary between the different samples owing to the very short structural distances responsible for the scattering in the *q* region. It must be noted that sample B, in comparison to sample A, shows sharp peaks which can be attributed to a finer nanocrystalline structure (more degree of homogeneity in the dimensions and distribution of the nanograins in the residual amorphous matrix), corresponding to the crystallization of α-Fe(Si) phase [14]. The evaluation of the crystalline phase formed with these treatments was made by means of subtraction of the amorphous halo of the pattern of the as-cast sample. The normalization to the total intensity constitutes the crystallinity ratio (χ), which is widely used to characterize semicrystalline materials [15]. As is expected, the obtained values of χ strongly depend on the annealing time. Accordingly, both samples exhibit clear peaks corresponding to the α-Fe(Si) phase. Table 1 provides numerical data on the partial crystalline phase and grain size deduced for the two samples from the XRD patterns. As can be seen, sample B (with larger annealing time than sample A) has a higher phase of nanocrystallization than sample A, which leads to a softer magnetic character as evidenced in the following results. 

**Figure 2:**
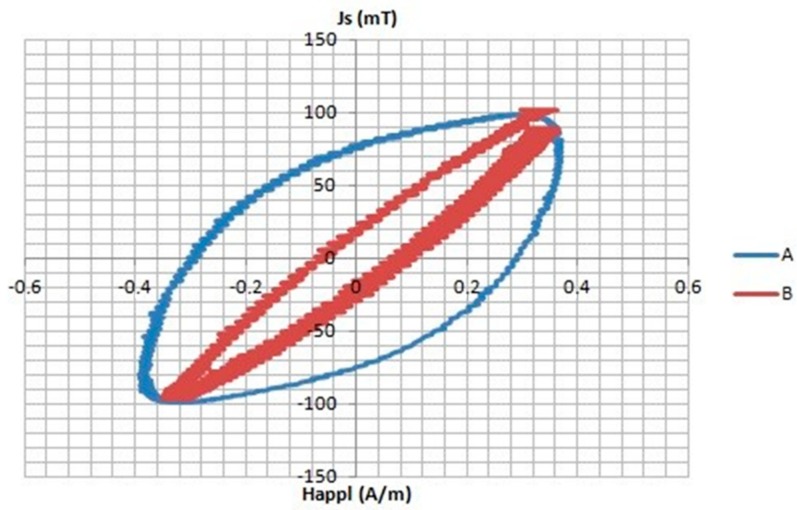
Hysteresis loop at low-applied magnetic field of the two nanocrystalline magnetic cores.

Figure 2 shows the hysteresis loops of the two samples at very low applied magnetic field (Rayleigh area). As can be seen, sample A presents a harder magnetic character compared to that of sample B, which indicates that the nanocrystalline character of sample B provokes a more effective intergranular coupling by exchange interaction leading to very soft magnetic behavior.

**Figure 3:**
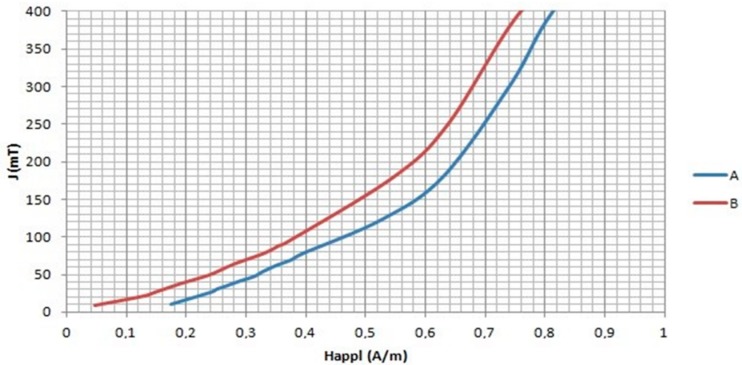
Magnetization curve, at low applied magnetic field, of the two nanocrystalline cores.

The low field region of the virgin magnetization curves is shown in Figure 3 for the annealed cores. Again, the curve of sample B indicates softer magnetic behavior in agreement with the XRD results.

On the other hand, within the field range, 0 < *H_appl_* < *H*_0_, these curves may be described by the so-called Rayleigh law for polarization *J*

(1) $$J=aH_{appl}+bH_{appl}^{2}$$

where the field *H*_0_ denotes the upper limit of the Rayleigh region. As is illustrated in Figure 3, the function *J*/*H_appl_* vs. the applied external field, *H_appl_*, leads to a linear relation in the Rayleigh region. The initial susceptibility, *χ_i_*= lim d*J*/d*H_appl_*, corresponds to the intercept on the ordinate being the coefficient of Equation (1), while the Rayleigh constant, b, should be the slope of the function *J*/*H_appl_*. The initial magnetic susceptibility and Rayleigh constant are parameters depending on the properties of ferromagnetic materials [16].

**Figure 4:**
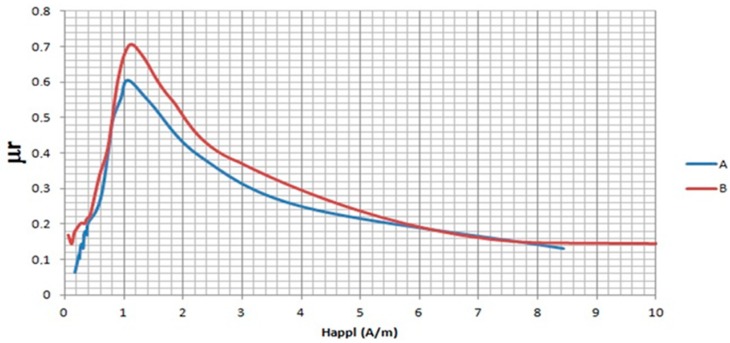
Relative permeability μ_r_ = *J/H*_0_ vs. the applied external field, *H_appl_*, for the two nanocrystalline samples.

It is interesting to note that the maximum of the curves of Figure 4 for the applied magnetic field is around 1 A/m. This value could be assigned to *H*_0_. Therefore, a linear region (0 < *H_appl_* < *H*_0_) corresponds to the Rayleigh region. Values of coefficient (a) (higher in core A) and coefficient (b) (the slope of the linear region, being large in core B) of Equation (1) for the two investigated cores could indicate a softer magnetic character of core B. Therefore, low-field magnetization curves of the two nanocrystalline cores can be accurately approximated by the Rayleigh model for the range of core A and core B, respectively.

**Figure 5:**
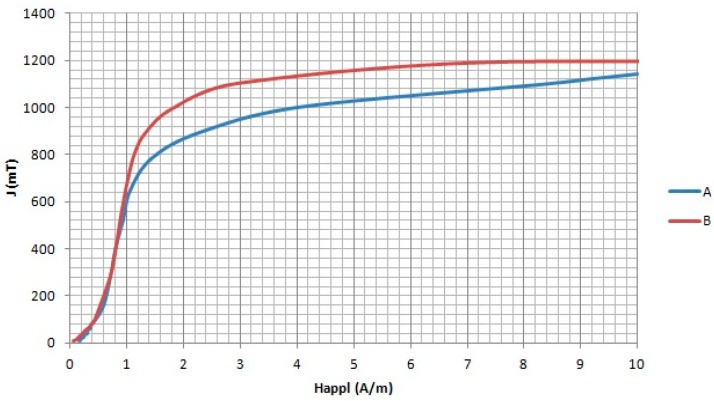
Virgin magnetization curve of the two nanocrystalline magnetic cores.

The virgin magnetization curves as a function of the external magnetic field are shown in Figure 5. Note that the approach to magnetic saturation is reached by core A (less volume of nanocrystals) with a large applied magnetic field as compared to core B.

Regarding the magnetization mechanism, it is assumed that the low-field magnetization (Rayleigh region) occurs by reversible displacement of 180°-Bloch domain walls within the regions of low applied magnetic field. The motion of Bloch walls, bound in each grain to the easy direction remaining active after demagnetization, is assumed. This effect should be more prominent in B core, where the partial volume of the nanocrystalline phase is larger than in A core leading to a more effective intergranular exchange coupling with a lower coercive field as can be seen from Figure 2. 

In fact, the suppression of magnetocrystalline anisotropy in this type of soft magnetic material has been attributed to an averaging process linked to the randomly oriented grains, which are ferromagnetically coupled by exchange interactions. Such averaging out of the crystalline anisotropy is a necessary condition for the achievement of good soft magnetic properties and, in particular, a high value of initial permeability [7]. The domain wall displacement seems to be the dominant mechanism of the magnetization process. Since the nanograins are gradually decoupled with decreasing partial volume of the nanocrystals, the weakened coupling results in an increase of the effective crystalline anisotropy, i.e., the domain structure changes from wide domains to a pattern of small irregular domains. As a consequence, domain wall displacement at the grain boundaries starts at low fields (propagation). The associated local hysteresis loops are described by means of the Rayleigh law and the macroscopic behavior of the material, observed either under alternating or rotational fields, is calculated in terms of the linear combination of such loops, according to the spatial distribution of the active easy axes.

This suggestion is also in agreement with a very low value of coercivity in comparison with amorphous sample. This effect is probably caused by the processes of nanocrystalline transformation, where the relief of internal stresses occurs, which decreases the magnetoelastic anisotropy and the number of regions with braked domain wall (DW) movement. Simultaneously, the thickness of DW increases to be much higher than the dimensions of nanocrystallites (10 nm) with the fact that the effective exchange correlation length (around 35 nm [7]) must positively determine such movement.

## 4. Conclusions

We studied the structural and, magnetic behavior of two toroidal cores based on nanocrystalline Finemet alloy. The nanocrystalline structure was developed by annealing (550 °C, 30 and 60 min) the core whose precursor state exhibited amorphous character. 

The X-ray diffraction paths of both treated cores reflect nanocrystalline character with a relative volume of nanograins in the sample treated for 60 min. As a consequence, this core presents softer magnetic behavior compared with the core treated for 30 min. The magnetic properties were studied in the low applied magnetic field (Rayleigh region) and analyzed in terms of the main mechanisms invoked to explain the magnetization process (displacement of domain walls for the amorphous and annealed cores. It is important to remark that the frequency of 100 Hz defines two regions for these dependencies in all cores which implies different microstructural mechanisms to explain the excess of eddy-current loss at high frequency range.

## Figures and Tables

**Table 1:** Numerical data on the partial crystalline phase and grain size.

Core	Crystallinity (%)	2θ_m_	D (nm)
A	67.2	45.200	13.5
B	75.4	45.200	14.9
